# Simultaneous alteration of residues 279 and 284 of the VP2 major capsid protein of a very virulent Infectious Bursal Disease Virus (vvIBDV) strain did not lead to attenuation in chickens

**DOI:** 10.1186/s12985-014-0199-7

**Published:** 2014-11-25

**Authors:** Nawel Ben Abdeljelil, Neila Khabouchi, Selma Kassar, Khaled Miled, Samir Boubaker, Abdeljelil Ghram, Helmi Mardassi

**Affiliations:** LR11IPT01 Laboratory of Molecular Microbiology, Vaccinology and Biotechnology Development, Université de Tunis El Manar, Institut Pasteur de Tunis, 13, Place Pasteur BP 74, 1002 Tunis Belvedere, Tunis Tunisie; Laboratoire d’Anatomie Pathologique Humaine et Expérimentale, Université de Tunis El Manar, Institut Pasteur de Tunis, 13, Place Pasteur BP 74, 1002 Tunis Belvedere, Tunis Tunisie; LR11IPT03 Laboratory of Veterinary Microbiology, Institut Pasteur de Tunis, 13, Place Pasteur BP 74, 1002 Tunis Belvedere, Tunis Tunisie

**Keywords:** vvIBDV, Infectious clone, *In vivo* reverse genetics, Mutagenesis, Tropism, Virulence, Attenuation

## Abstract

**Background:**

Cell culture adaptation of very virulent infectious bursal disease virus (vvIBDV) was shown to be mainly associated with the VP2 capsid protein residues 253, 279, and 284. The single mutation A284T proved critical for cell culture tropism, but did not confer efficient virus replication, which at least required one additional mutation, Q253H or D279N. While the double mutation Q253H/A284T was unambiguously shown to confer both efficient replication in cell culture and attenuation in chickens, conflicting results have been reported regarding the replication efficiency of vvIBDV mutants bearing the D279N/A284T double mutation, and no data are hitherto available on their virulence in chickens.

**Findings:**

Here we used an *in vivo* reverse genetics system to assess the impact of the D279N/A284T double mutation on the replication and attenuation of a chimeric IBDV virus, whose polyprotein derived from a non-culturable vvIBDV clinical isolate. We found that the D279N/A284T double mutation did indeed confer efficient replication in chicken embryo fibroblast (CEF) cell culture, but the mutant virus remained highly pathogenic to chickens.

**Conclusions:**

The double mutation D279N/A284T of the VP2 major capsid protein of vvIBDV is sufficient to confer cell culture tropism and replication efficiency, but does not necessarily lead to virus attenuation.

## Background

Infectious bursal disease virus (IBDV), a member of the family *Birnaviridae*, genus *Avibirnavirus*, is the causative agent of an avian restricted disease known as “Gumboro disease” [1]. The virus causes severe immunodepression in young chicken by destruction of B cells and finally the bursa of Fabricius [2]. These infected immunodepressed chickens become highly susceptible to secondary infections and have a reduced capacity to respond to vaccination [3-5]. IBDV is therefore a major economic issue for the poultry industry. Protection of susceptible flocks is achieved by vaccination against IBDV using either attenuated live or inactivated virus [6].

The genome of IBDV consists of two segments of double-stranded RNA (dsRNA), referred to as segments A and B [7,8]. The smaller segment B (2.8 kbp) harbours a single open reading frame (ORF) encoding VP1, a protein with an RNA-dependent RNA polymerase and capping enzyme activities [9-11]. Genome segment A (3.2 kbp) contains two overlapping ORFs. The first smallest ORF encodes VP5, a 17-kDa non structural protein, while the second ORF codes for the precursor polyprotein NH2-pVP2-VP4-VP3-COOH [12,13]. This polyprotein is co-translationally self-cleaved, yielding ultimately the capsid protein VP2, the viral protease VP4, and the multifunctional scaffolding nucleocapsid VP3 [14-16].

Two different IBDV serotypes exist, serotypes 1 and 2 [17]. Wild-type isolates belonging to serotype 1 are pathogenic for chickens, while serotype 2 isolates, which are mainly recovered from turkeys, are regarded as non pathogenic for chickens [18,19]. IBDV serotype I includes four pathotypes, namely attenuated, classical virulent, antigenic variant, and very virulent (vvIBDV). The latter pathotype has emerged in Europe since the mid 1980s, and was since reported worlwide as the deadliest pathotype, with 60–100% mortality in chickens [20-25].

Early studies using site-directed mutagenesis coupled with reverse genetics showed that capsid VP2 amino acid residues at positions 253, 279, and 284, are involved in cell culture adaptation and/or virulence of vvIBDV [26-30]. More recently, the contribution of residues 249 and 265 was reported [31]. It was shown that simultaneous alteration of residues 253 (Q to H) and 284 (A to T) confers cell culture tropism and efficient replication in chicken embryo fibroblast (CEF) cells, but neither of the two mutations could do so when introduced individually [27,28]. According to the same independent studies, the attenuating phenotype of the double exchange Q253H and A284T was unambiguously demonstrated. It has also been shown that the double mutation D279N and A284T results in cell culture adaptation of the vvIBDV strain HK46, which grew to high titers [29]. This finding could not be confirmed with vvIBDV strain UK661, since the molecularly engineered virus bearing the double mutation D279N/A284T yielded very low titers compared to that bearing the double exchange Q253H/A284T [28]. Furthermore, with both HK46 and UK661 strains, the impact on virulence of the double mutation D279N/A284T has not been explored.

Here using a chimeric virus generated by *in vivo* reverse genetics, we show that the double exchange D279N/A284T in the VP2 sequence of vvIBDV indeed confers efficient replication in CEF cells, but could not attenuate the rescued chimeric virus in chickens.

## Materials and methods

### Cells

Primary Chicken Embryo Fibroblast (CEF) cells were freshly prepared from 9 to 11-day-old-embryonated specific pathogen-free (SPF) eggs. The cells were grown in Dulbecco’s minimal essential medium (DMEM) supplemented with 10% foetal bovine serum (FBS) at 37°C in a humidified 5% CO2 incubator. CEF cells were used for transfections, virus propagation and tittering.

### Plasmid constructs

Plasmid constructs pVAXSA.Rib and pVAXSB.Rib [32,33] contain the full-length cDNA sequence of the cell culture-adapted and attenuated P2 strain segments A and B, respectively [34]. In both constructs, the 5′ end of each genomic segment was fused to the transcription start site of the immediate early CMV promoter, while a hepatitis delta virus (HDV) ribozyme sequence was added at the 3′ end. This cloning strategy was designed so that the host RNA polymerase II will produce segments A and B transcripts with authentic 5′ and 3′ termini; an *in vivo* reverse strategy which has significantly improved the titer of rescued IBDV [32].

### Construction of chimeric and mutant infectious IBDV cDNA clones

The full-length coding sequence of the polyprotein and the 3′ non-coding region of PO7, a Tunisian bursal-derived field isolate of vvIBDV [35], was amplified by PCR using the primer pair IB2SP1/SAR1 (Table 1) and the Expand High Fidelity *Taq* polymerase mix (Roche Applied Science, Germany). The resulting amplicon was cloned into *kpn*I/*Eco*RI-restricted pCR2.1 plasmid vector (Invitrogen), yielding the construct pCR.PO7poly.wt. This was next used as template to substitute the VP2 amino acid residues aspartic acid (D) to asparagine (N), and alanine (A) to threonine (T), at positions 279 and 284, respectively. Site-directed mutagenesis was carried out by overlap extension using PCR [36]. Briefly, two independent PCR reactions were performed using the primer pairs IB2SP1/MutR and MutS/SAR2. MutR and MutS are the mutagenic overlapping primers (Table 1). The two PCR products were combined, denatured, annealed, and subjected to 3′ extension followed by a fusion PCR amplification using the external primer pair IB2SP1/SAR2. The resulting PCR product was restricted with *Kpn*I/*Sal*I and subcloned into the similarly digested pCR.PO7poly.wt plasmid, yielding pCR.PO7poly.mt. Effective incorporation of the nucleotide changes was confirmed by DNA sequencing.

**Table 1:** **Primers used in PCR amplifications, cloning and mutagenesis of IBDV segments A and B**

**Primer designation**	**Sequence (5′-3′)**	**Orientation**	**Location**	**Restriction site**
IB2SP1	CAT*GGTACC*ATGACAAACCTGCAAGATCAAACC	+	131-154	*Kpn*I
SAR1	AGA*GAATTC*AGGGGACCCGCGAACGGATCCAATT	-	3237-3261	*Eco*RI
MutS	GCA**A**ACAATGGGCTAACG**A**CCGGCACTGACAACC	-	962-995	-
MutR	GG**T**CGTTAGCCCATTGT**T**TGCGGCCACAGCTCTG	-	949-982	-
SAR2	CAAGAATCCC*GTCGAC*TACG	-	1739-1720	*Sal*I
SAS1	GGATACGATCGGTCTGACCC	+	1-20	-
SBS1	GGATACGATGGGTCTGACCC	+	1-20	-
SBR1	GGGGCCCCCGCAGGCGAAGG	-	2826-2807	-

Restriction enzyme sites are italicized.

Nucleotides used for mutagenesis of VP2 residues 279 and 284 are indicated in bold.

**Figure 1:**
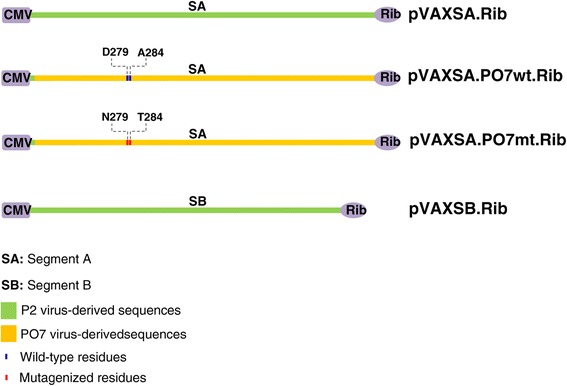
**Schematic representation of IBDV segments A and B infectious cDNA constructs used in the present study.**

Next, pCR.PO7poly.wt was restricted with *Bsp*EI/*Eco*RI and subcloned into *Bsp*EI/*Eco*RI-digested pVAXSA.Rib. Since *Eco*RI digestion of pVAXSA.Rib eliminates the HDV ribozyme sequence, the latter was restored at the 3′ end of PO7 segment A, using the same previously described strategy [32]. The resulting plasmid construct, pVAXSA.PO7wt.Rib (Figure 1), contains a full-length chimeric segment A cDNA sequence where the 5′ end first 215 nucleotides originated from the attenuated P2 strain, while the remaining sequence (216 to 3261) derived from the Tunisian PO7 vvIBDV strain.

Finally, the plasmid pCR.PO7poly.mt was cleaved with *Bsp*EI/*Sal*I and inserted into the corresponding restriction sites of pVAXSA.PO7wt.Rib, yielding pVAXSA.PO7mt.Rib, which contains the double mutation D279N/A284T in the PO7 VP2 capsid sequence (Figure 1).

### Rescue of IBDV via *in vivo* reverse genetics

To rescue chimeric IBDV virus expressing the wild-type or the D279N/A284T mutated PO7 polyprotein, 5 μg each of plasmid constructs pVAXSA.PO7wt.Rib or pVAXSA.PO7mt.Rib were cotransfected with the same amount of pVAXSB.Rib. For comparative purposes, pVAXSA.Rib and pVAXSB.Rib plasmid constructs were cotransfected in parallel in order to rescue the cell culture-adapted and attenuated P2 strain, as previously described [32]. Co-transfections of CEF cells growing at 80% confluence in 100-mm culture dishes were performed using FuGENE-6, according to the manufacturer’s protocol (Roche Applied Science). At 3 days post-transfection, while cytopathic effect was evident, the supernatant was collected, clarified by low-speed centrifugation, aliquoted and then frozen at −70°C. This original unpassaged virus stock (P_0_) was further subjected to two consecutive passages (P_1_ and P_2_). The titers of the original virus stock (P_0_) and the two passages (P_1_ and P_2_) were determined upon three independent assays on CEF cells and expressed as TCID_50_/ml (Tissue Culture Infectious Dose 50).

### Evaluation of the rescued IBDV virus pathogenicity in chickens

Three-week-old specific-pathogen-free (SPF) chicks were used. A group of 30 chickens were raised in isolators with filtered air-flow and randomly divided into three groups of 10 chicks each. Prior to inoculation, chickens were checked for seronegativity to IBDV. To minimize the risk that viruses used to infect chickens may contain undesired mutations in either genomic segment, only the unpassaged (P_0_) virus stock (the supernatant of cotransfected CEF cells) of the rescued viruses was used. Chickens were inoculated with 10^5^ TCID_50_ of the P_0_ stock of each rescued virus, or with phosphate-buffered saline (PBS), by eye drop.

Clinical signs were determined using a newly developed symptomatic index score (0 to 3) as described in Nouen et al. [37]. Chickens were humanely euthanized at day 10 post-inoculation. Bursas and spleens were removed, weighed, and the mean organ/body weight (BW) ratio was determined according to Ismail and Saif [38] using the following formula: organ weight in grams × 1000)/BW in grams.

Organs were split into two parts. One part was used to extract total RNA, and the second part was processed for histopathology.

### Determination of the nucleotide sequence of the genomic segments A and B of IBDV recovered from the bursas of infected chicks

Bursas were cut into small pieces, ground with sterile sea sand in PBS, and the homogenates were centrifuged at full-speed in an eppendorf centrifuge for 10 min at +4°C. RNA was extracted using the QIAmp viral RNA mini kit (Qiagen) following the manufacturer’s instructions. RT-PCR amplifications were performed using the Qiagen OneStep RT-PCR-Kit (Qiagen), essentially as recommended by the supplier, using segment-specific primer pairs SAS1/SAR1 and SBS1/SBR1 (Table 1). PCR products were cloned into pCR2.1 plasmid vector and subjected to nucleotide sequencing using additional sense and reverse primers. The full-length sequence of both segments was determined with the exception of the first and last 20 nucleotides (primer binding sites). For each segment, the nucleotide sequence was confirmed twice.

### Histopathological examination

Formalin-fixed bursa were embedded with paraffin, sectioned and stained with haematoxylin/eosin following standard procedures. Depending on the severity of bursal follicular necrosis, bursal lesions were assigned the score (BLS) of 0 (normal), 1 (mild lymphoid depletion with absence of necrosis/oedema), 2 (moderate lymphoid depletion along with focal necrosis and interfollicular oedema), 3 (severe lymphoid depletion virtually leaving no lymphocyte but only reticular cells and proliferating fibrous tissue, or 4 (atrophy of follicles usually with cystic spaces, infolding of epithelium and marked fibroplasias).

### Statistical analysis

Statistics were calculated using Prism software (GraphPad Software Inc.). The data were presented as the means and evaluated by one-way ANOVA followed by Newman-Keuls multiple comparison test. For comparison of virus titers, the unpaired *t* test was used. The level of significance was *P* <0.05.

## Results

### *In vivo* reverse genetics mediated recovery and replication efficiency in CEF cells of IBDV specifying the polyprotein sequence of PO7, a Tunisian vvIBDV strain

**Figure 2:**
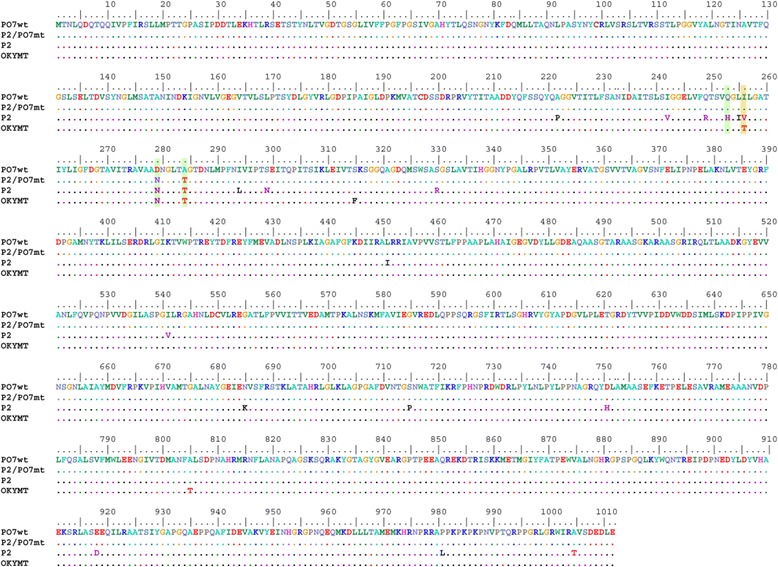
**Amino acid sequence alignment of the polyprotein sequences of the Tunisian vvIBDV wild-type PO7 strain (PO7wt), the cell culture-adapted and attenuated P2 and OKYMT IBDV strains, and the cell culture-adapted, but virulent, D279N/A284T mutated P2/PO7 chimeric IBDV virus (P2/PO7mt).** Amino acid residues (positions 253, 279, and 284) known to be involved in cell culture adaptation and virulence are highlighted in green. The residue 256 which could have contributed to OKYMT attenuation is highlighted in orange.

**Figure 3:**
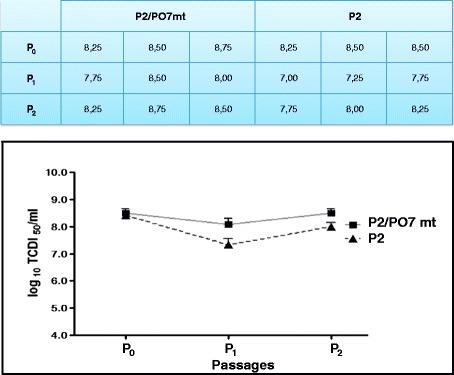
**Titers in CEF cells of the rescued P2 and P2/PO7mt molecularly engineered IBDV viruses.** The table on top shows the titers (in TCID50/ml) of the rescued viruses obtained after three independent titering assays from cotransfected CEF cells (P_0_), and after two sucessives passages (P_1_ and P_2_). The corresponding graph is shown on bottom.

Using as recipient hosts infectious clones pVAXSA.Rib and pVAXSB.Rib of the cell culture-adapted and attenuated IBDV P2 strain, we attempted to rescue in CEF cells IBDV virus specifying the polyprotein sequence of the Tunisian bursal-derived vvIBDV PO7 strain [35]. The VP2 amino acid residues at positions 253 (Q), 279 (D), and 284 (A) of the wild-type PO7 strain corresponded to those of non culturable vvIBDV strains. For this purpose, we exchanged the P2 segment A sequence extending from the natural *Bsp*EI restriction site (nucleotide position 215) to the 3′ end ultimate nucleotide with its corresponding sequence of the wild-type Tunisian PO7 vvIBDV strain. With this cloning strategy, the entire polyprotein amino acid sequence specified by this chimeric P2/PO7 segment A is identical to that of the wild-type PO7 strain, and differs from the segment A cDNA infectious clone of the P2 strain by 19 amino acid residues (P222A, V242I, R249Q, H253Q, I255L, V256I, N279D, T284A, L294I, N299S, R330S, I451L, V541I, K685N, P715S, H751D, D918E, L981P, and T1005A) (Figure 2). As previously shown, the IBDV P2 strain which bears the VP2 amino acids H253, N279, and T284, common to all tissue-culture-adapted IBDV strains, was readily rescued when CEF cells were co-transfected with pVAXSA.Rib and pVAXSB.Rib plasmid constructs [32]. Indeed, cytopathic effect typical of IBDV replication could be observed as soon as three days post-transfection, and was successfully passaged twice, using the supernatant of transfected cells. No cytopathic effect could be observed when the chimeric P2/PO7 segment A harbouring the wild-type PO7 polyprotein sequence (bearing the amino acid combination Q253, D279, and A284) was used in co-transfection experiments (pVAXSA.PO7wt.Rib and pVAXSB.Rib), and no signs of virus replication could be detected even after three successive passages (data not shown). By contrast, when the segment A infectious cDNA clone bearing the double mutation D279N/A284T in the PO7 polyprotein sequence (pVAXSA.PO7mt.Rib) was used in cotransfections, a cytopathic effect was apparent and could be passaged twice. Upon cotransfections, the rescued chimeric mutated P2/PO7 (P2/PO7mt; bearing the amino acid combination Q253, N279, and T284) and P2 viruses yielded nearly equivalent titers, varying from 10^7^ to 10^8.5^ TCID50/ml. Through passages 1 and 2, the P2/PO7mt virus displayed approximately 0.5 log higher titers than P2, but these differences in titers was not statistically significant (*P* = 0.266) (Figure 3).

### Evaluation of the pathogenicity of the rescued chimeric P2/PO7 IBDV in SPF chickens

Chickens inoculated by eye drop with 10^5^ TCID50 of unpassaged P2/PO7mt virus stocks (using the supernatant of CEF transfected cells) showed typical clinical symptoms of vvIBDV infection. Nine of ten chickens (90%) died in the first 3 days post-inoculation. The bursa/body and spleen/body weight ratios (B/BW and S/BW, respectively) were significantly lower than in normal chickens (*P* <0.05; one-way ANOVA followed by Newman-Keuls multiple comparison test) (Table 2). By contrast, none of the P2-inoculated chickens showed typical clinical signs of vvIBDV infection during the whole 10-day observation period, and all chickens of this group survived. The bursa/body and spleen/body weight ratios were not significantly different from normal chickens (*P* >0.05).

**Table 2:** **Pathogenicity of the rescued P2 and P2/PO7 molecularly engineered IBDV viruses in SPF chicken**

**Virus**	**Group** ^**a**^	**Score** ^**b**^	**B/BW ratio** ^**c**^	**S/BW ratio** ^**d**^	**Dead** ^**e**^
P2/PO7	1	3	2.936	1.834	9/10
P2	2	0	4.788	1.959	0/10
-	Control	0	7.024	2.041	0/10

^a^Groups of 10 SPF chickens each were inoculated at the age of 3 weeks with either P2 virus or P2/PO7 mutant virus collected from the supernatant of transfected CEF cells. Chickens in the control group were inoculated with PBS.

^b^Score 0: no damage; score 1: mild damage; score 2: moderate damage; score 3: severe damage.

^c^B/BW: (average bursa in grams · 1000)/total body weight in grams. Values show the averages for 10 chickens.

^d^S/BW, (average spleen in grams · 1000)/total body weight in grams. Values show the averages for 10 chickens.

^e^Number of dead chickens at 3 days post-inoculation.

### Histopathological examination of the bursa

**Figure 4:**
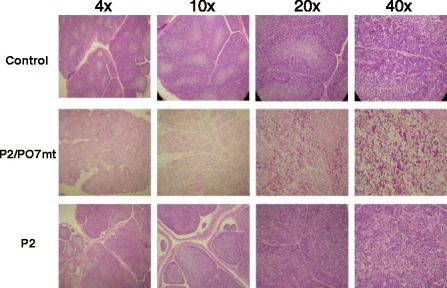
**Histopathological appearance of hematoxylin/eosin sections of bursa.** Note the severe necrosis, atrophy, and loss of the follicular architecture in sections derived from bursa of P2/PO7-infected chickens. By contrast, histopathologic sections of bursa derived from the control or P2-infected chikens showed normal follicles and follicular connective tissues.

All bursae of dead chicks inoculated with the resuced P2/PO7mt IBDV virus showed severe necrosis, atrophy, and loss of the follicular architecture. By contrast, histopathologic sections of bursa derived from control (inoculated with PBS) or P2-infected chicks were similar and had normal follicles and follicular connective tissues (Figure 4). These results clearly indicate that the molecularly engineered cell culture-adapted P2/PO7mt IBDV was not attenuated in chickens.

### Sequence analysis of segments A and B extracted from the bursae of dead chicks

Sequence analysis of both genomic segments of the rescued P2/PO7mt virus from the bursa of Fabricius of dead chicks did not reveal any amino acid change (data not shown).

## Discussion

Previously we reported the nucleotide sequence of the VP2 hypervariable region of bursal-derived Tunisian vvIBDV field isolates and the polyprotein coding sequence of PO7, a Tunisian vvIBDV representative isolate [35]. This strain was recovered from the bursa of dead chickens from a Tunisian layer flock that have experienced a severe outbreak with nearly 100% motality. Experimental inoculation of SPF chicks with the original bursal homogenate of PO7 strain killed all the inoculated birds in the first three days. Birds that succumbed to infection showed inappetence and diarrhea. Autopsy of dead chicks showed mainly haemorrhagic and atrophied bursa, as well as petechial haemorrhages on the mucosa and in muscular tissues. The very virulent pathotype of the Tunisian PO7 strain was further confirmed molecularly, giving the presence of the four amino acid residues, A222, I256, I294, and S299 in the hypervariable region of its VP2 sequence [35].

Several attempts to adapt the PO7 isolate to cell cultures proved unsuccessful. This result was congruent with the nature of its VP2 amino acid residues at positions 253 (Q), 279 (D), and 284 (A). Using an *in vivo* reverse genetics approach that we have previously developed in our laboratory [32,33], we constructed a chimeric IBDV infectious clone whose polyprotein derived from the Tunisian vvIBDV PO7 strain, using segments A and B infectious cDNA backbones of strain P2, a tissue culture-adapted and attenuated German IBDV strain.

The finding that the double exchange D279N/A284T yielded a high-titer virus further confirmed the results of Lim et al. [29], in that the two amino acid residues N279 and T284 are sufficient to confer cell culture adaptation. The inability to obtain high-titer virus with the vvIBDV UK661 bearing the same residues may stem from differences in the strain’s genetic background, or could be due to the approach of reverse genetics that has been used [28]. Indeed, Lim et al. [29] used an *in vivo* reverse genetics method, which could have contributed to rescue high-titer virus. We also used *in vivo* reverse genetics, but unlike the approach of Lim et al. [29], our method has been conceived to generate genomic transcripts with authentic 5′ and 3′ ends [32], which is likely to be the main feature that allowed us to rescue high-titer chimeric P2/PO7 mutated virus.

The data obtained in this study demonstrate, for the first time, that acquisition of cell culture tropism by IBDV was not accompanied with its attenuation in chickens. The D279N/A284T double exchange in the VP2 capsid protein proved sufficient to confer cell culture adaptation and replication efficiency but did not lead to attenuation. This finding is in agreement with the study previously reported by Raue et al. [39]. Indeed, they noticed that reversion of the T284 substitution back to the wild-type phenotype (A284) was not accompanied with increased virulence, the full recovery of which was concomitant with reversion of the H253 residue to the wild-type (Q253), which occurred 10 to 11 days after. Sequencing of segments A and B of the culture-adapted, but virulent, P2/PO7mt virus recovered from the bursa of dead chickens showed no new mutations and confirmed the presence of the virulence-associated Q residue at position 253, as well as the conservation of N279 and T284 residues. As mentioned above, in our study we infected chickens with unpassaged virus stocks (using the supernatant of transfected cells); therefore, we minimized the risk to generate new spontaneous mutations that might change the phenotype of the rescued mutant virus. Consistent with our sequencing results is the fact that P2/PO7mt virus recovered from dead chickens can still be propagated in cell culture.

However, the finding that the cell culture-adapted P2/PO7 mutant virus remained pathogenic contrasts with the attenuated nature of strain OKYMT [40], which like P2/PO7mt displays the amino acid combination Q253, N279, and T284 (Figure 2). Comparative sequence analysis revealed that OKYMT differed from the mutant P2/PO7 virus at the three amino acid positions, 256, 315, and 805 (Figure 2). Strikingly, the amino acid residue at position 256 has been reported to be involved in the replication efficiency and virulence of IBDV [31]. Indeed, a mutant IBDV whose Isoleucine residue at position 256 was changed to Valine proved less virulent. Unlike P2/PO7mt virus, which bears an Isoleucine residue at position 256 (I256), the attenuated OKYMT strain shows a Threonine at the same position (T256). Hence, one can wrightfully argue that the T256 residue could have contributed to OKYMT attenuation. However, one should not dismiss the possibility that amino acid positions 315 and, to a lesser extent, 805 could have further attenuated OKYMT strain. Moreover, attenuation of OKYMT involved 43 successive passages (20 passages in chorioallantoic membrane, 15 in yolk sac, and 8 in CEF cells), which could have resulted in the accumulation of attenuating mutations in the polymerase VP1 gene encoded by segment B. Indeed, it is now well established that segment B contributes to the virulence of vvIBDV [41-43]. It has been shown that a single change in VP1 sequence contributes even more to attenuation than a substitution in the polyprotein sequence [41]. Consistent with this finding, it was shown that a Valine to Isoleucine substitution in VP1 at amino acid position 4 attenuates vvIBDV in chickens, but increased its replication in CEF cells [42]. Sequence comparison of VP1 genes of P2 (specified by our chimeric P2/PO7mt virus) and the attenuated OKYMT would provide a more thorough analysis. Unfortunately, only the segment A sequence of OKYMT has been published hitherto.

## Conclusions

Based on the work described here, we conclude that the double mutation D279N/A284T of the VP2 major capsid protein of vvIBDV is sufficient to confer cell culture adaptability and replication efficiency, but it does not necessarily lead to virus attenuation.
